# Economic outcomes associated with acute interstitial pneumonia in Central U.S. High Plains feedyards

**DOI:** 10.1093/tas/txaf091

**Published:** 2025-07-11

**Authors:** Merri E Day, Dustin L Pendell, Brad J White, Phillip A Lancaster, Robert L Larson

**Affiliations:** Texas A&M AgriLife Extension Service, Department of Agricultural Economics, Texas A&M University, Amarillo, TX 79106, USA; Department of Agricultural Economics, College of Agriculture, Kansas State University, Manhattan, KS 66506, USA; Beef Cattle Institute, Kansas State University, Manhattan, KS 66506, USA; Beef Cattle Institute, Kansas State University, Manhattan, KS 66506, USA; Department of Clinical Sciences, College of Veterinary Medicine, Kansas State University, Manhattan, KS 66506, USA; Beef Cattle Institute, Kansas State University, Manhattan, KS 66506, USA; Department of Clinical Sciences, College of Veterinary Medicine, Kansas State University, Manhattan, KS 66506, USA; Beef Cattle Institute, Kansas State University, Manhattan, KS 66506, USA; Department of Clinical Sciences, College of Veterinary Medicine, Kansas State University, Manhattan, KS 66506, USA

**Keywords:** AIP, cattle, estimated net return, expected net return, respiratory disease, treatment outcome

## Abstract

The objective of this study was to evaluate net returns for Central U.S. high plains feedyard cattle identified with acute interstitial pneumonia (AIP) ante-mortem and postmortem (*n* = 5,339) and to examine economic outcomes across sex, placement weight, and number of AIP treatments. A decision tree framework was implemented to estimate net returns of cattle identified with AIP, where decision nodes represented choices made by the producer, and branches represented potential outcomes following a decision. The initial decision node was whether to treat cattle for AIP after the first identification for illness or to sell (cull) soon after diagnosis at reduced weight and price compared to cattle in the cohort sold at finished weight. Following initial treatment, cattle that remained in the feedyard either finished (with or without further treatment), were culled, or died after additional diagnosis and treatment. Probabilities of incidents at each node were obtained from the data distribution. This research indicates that estimated net returns for feedyard cattle identified with AIP vary by sex, placement weight, and number of AIP treatments. The expected net return to feeding healthy cattle was $193.67/animal, while the expected net return for cattle that finished after AIP treatment was -$639.71/animal for cattle treated once for AIP, -$612.41/animal for those treated twice for AIP, and -$529.57/animal for those treated three or more times for AIP. However, other health indicators and risk factors not included in this analysis should be considered when deciding whether to keep or cull feedyard cattle identified with AIP.

## Teaser

Findings from analysis indicate that expected net returns from feedyard cattle identified with acute interstitial pneumonia (AIP) are considerably lower than the expected net return for healthy cattle. However, estimated returns remain positive for cattle that recover and finish with their cohort, regardless of sex, placement weight, or number of AIP treatments.

## Graphical Abstract

**Graphical Abstract:**
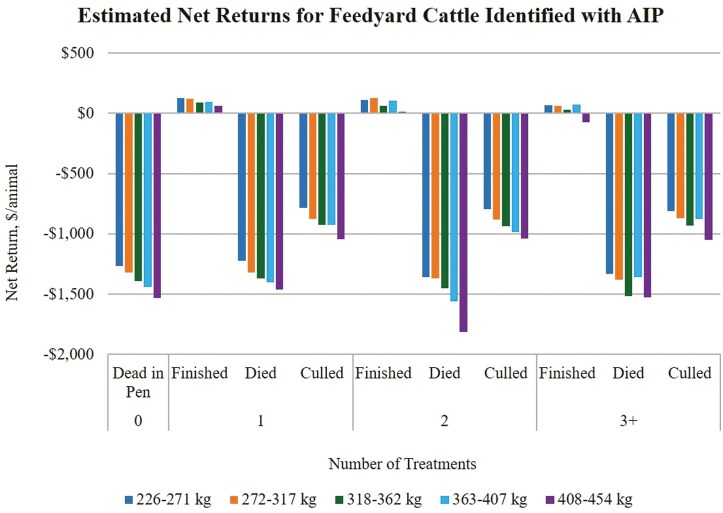

## INTRODUCTION

Research indicates health outcomes are correlated with animal performance (Irsik et al., 2006; Belasco, Cheng & Schroeder, 2015). Agricultural economists have also linked cattle health and performance to net returns (Mark, Schroeder, & Jones, 2000; Brooks et al., 2011). Further research has shown that timing of disease events and number of treatments also impact net return to cattle feeding (Babcock et al., 2010; Davis-Unger et al., 2017). Economic costs may stem from death loss due to treatment failure, foregoing culling for treatment, or culling cattle that would have finished profitably. Common feedyard diseases such as respiratory illnesses, though treatable, may have sizable adverse effects on health and economic returns if not effectively managed.

Acute interstitial pneumonia (AIP) is a respiratory illness that affects approximately 3% of U.S. feedyard cattle (USDA APHIS, 2013) and has been identified as one of the most important feedyard respiratory diseases (Amosson et al., 2006). While other respiratory illnesses such as bovine respiratory disease (BRD) often affect cattle early in the feeding phase (<40 d on feed) (Griffin, 2014), AIP is typically identified later in the feeding phase (>45 d on feed) (Woolums, 2003, 2015). Since AIP often affects cattle near harvest, there is potential for great economic losses from investment in feed, yardage, and interest, in addition to the cost of the animal (Loneragan et al., 2001a). However, there is little research quantifying the economic impacts of AIP. The objective of this analysis was to estimate net returns for cattle identified with AIP ante-mortem and postmortem in feedyards and evaluate economic outcomes across sex, placement weight, and number of treatments.

## MATERIALS & METHODS

Data was collected from existing records maintained by cooperating operations; therefore, IACUC approval was not acquired for this study. An initial dataset was created using retrospective feedyard records and individual cattle health records collected from 9 Central U.S. high plains feedyards, along with economic information. Cohort level feedyard data included average placement weight, placement date, average finishing weight, harvest date, average daily gain, and average feed consumption. Cattle health data included diagnosis, date of treatment, weight at treatment, and outcome of treatment and was matched with cohort level feedyard data using cohort identification numbers. Economic information came from the Livestock Marketing Information Center and Federal Reserve Bank of Kansas City.

### Data Cleaning

**Figure 1.**
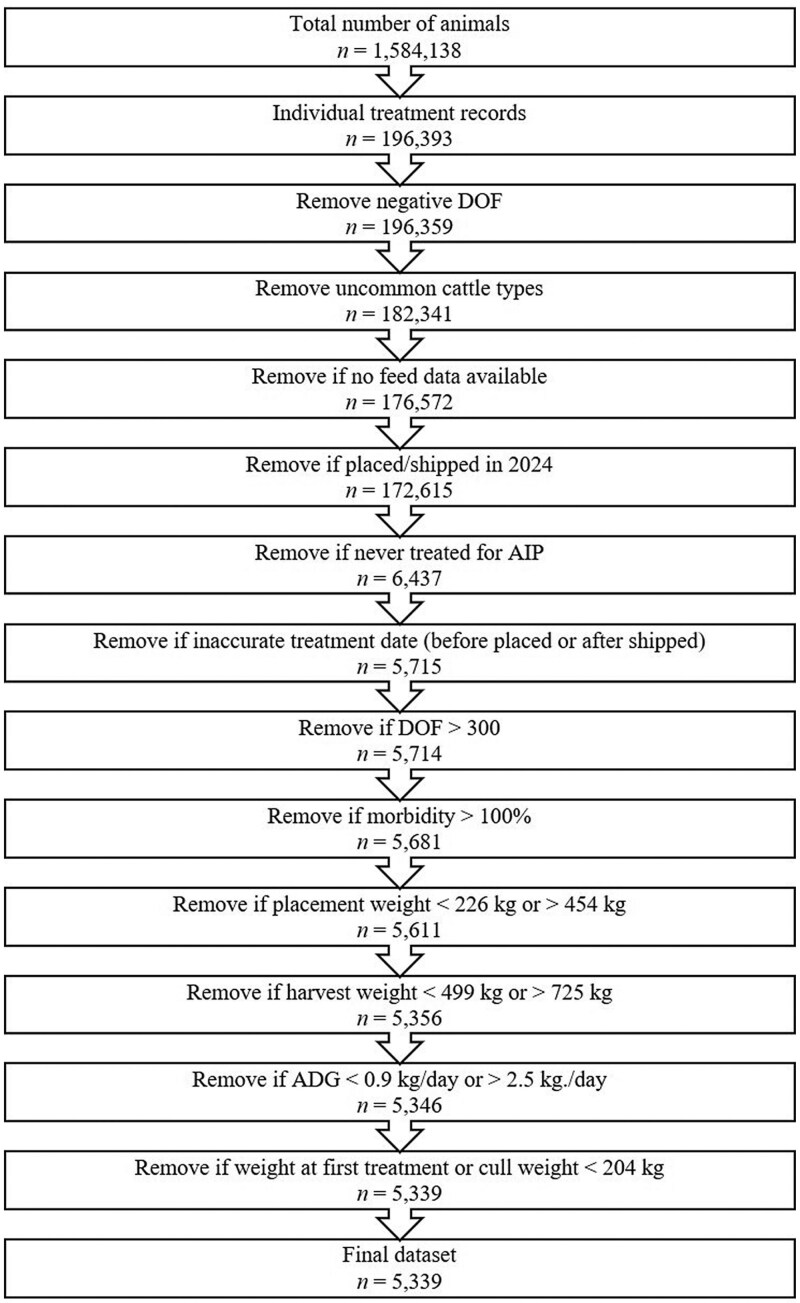
Data cleaning process to identify cattle with acute interstitial pneumonia (AIP) in health and cohort records from 9 Central U.S. high plains feedyards from 2019 to 2023.

Inclusion and exclusion criteria for the final dataset are described in Figure 1. Exact copies of individual health records were considered duplicates and therefore omitted, leaving 196,393 unique observations. Observations with negative estimated days on feed (DOF) were removed. Uncommon cattle types (i.e., Holsteins and Mexican cattle) and observations from mixed-sex pens were removed from the dataset to allow investigation of the effect of sex. Observations for which feed data were unavailable were removed from the dataset. Cattle with placement dates or closing dates in 2024 were removed from the dataset, leaving beef steers and heifers placed in 9 Central U.S. high plains feedyards from 2019 through 2023.

AIP cases were identified by feedyard personnel using pre-defined criteria. These criteria included but were not limited to signs of respiratory distress, increased exhalation effort, increased clear nasal and/or ocular discharge, and rapid onset of severe clinical disease. Only AIP cases, which represented approximately 3.5% of treated cattle from the initial dataset, were evaluated in this study. These data were further refined by excluding those with unreliable treatment dates (treatment date reported as being prior to placement or after shipping). Cattle on feed for more than 300 d were considered outliers and excluded from the analysis. Observations with reported pen-level morbidity greater than 100% were removed. Cattle placed in the feedyard weighing less than 226 kg or more than 454 kg were excluded due to inconsistent availability of price records for lightweight and heavyweight cattle. For similar reasons, cattle finishing at less than 499 kg or greater than 725 kg were also excluded. Cattle weighing less than 204 kg at first AIP treatment or when culled were considered outliers and removed. The final dataset for this analysis included 5,339 feedyard cattle from 3,216 cohorts that were identified with AIP (Figure 1).

### Data Management

Days on feed (DOF) for each animal were determined by subtracting placement date from estimated harvest date. Placement weight, harvest weight, morbidity, and mortality were cohort-level recorded variables. Average daily gain (ADG) of the cohort was determined by dividing total weight gained during the feeding period by estimated DOF. Average feed conversion (AFC) of the cohort was determined by dividing dry feed consumption by total weight gained during the feeding period.

In the dataset, healthy cattle were defined as those having no record of a disease event in the feedyard, while treated cattle were defined as those receiving treatment following a disease event diagnosis. An AIP event was defined as having been treated for or died from AIP. Additional variables indicating AIP event outcomes were created for this analysis. The number of AIP treatments was determined by the number of events designated as AIP with a “Treatment” outcome. AIP events with a “Death” outcome without prior AIP treatment were indicated as having been found dead in the pen. Event outcomes beyond the first AIP treatment were defined as Death (died in the feedyard), Cull (culled prior to harvest), and Finish (finished with cohort). Deaths and Culls were not necessarily attributed specifically to AIP, but rather denoted outcomes following at least one AIP treatment.

### Data Analysis

**Figure 2.**
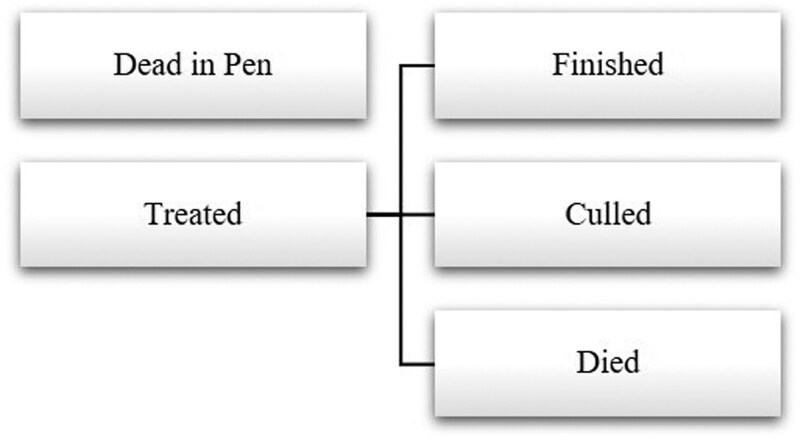
Example Decision Tree Framework. Dead in Pen = never treated for AIP; Treated = treated at least once for AIP; Finished = harvested with cohort; Died = died prior to harvest; Culled = culled prior to expected harvest.

A decision tree framework was implemented to estimate the net returns of cattle with various disease outcomes at multiple decision points. A decision tree framework includes decision nodes, which represent a choice made by the producer, and branches, which represent potential outcomes following a decision. Decision nodes were selected based on how feedyard managers make decisions following cattle treatment in the feedyard. As seen in Figure 2, the initial decision was whether or not to treat cattle for AIP after the first identification. Following initial treatment, cattle that remained in the feedyard may have finished with or without further disease events, culled or died after additional diagnosis and treatment.

Weekly feeder cattle prices and dressed carcass prices were collected from the Livestock Marketing Information Center (n.d). Dressed cull cow prices have been found to be significantly correlated with prices received for feedyard culls, and thus were used as a proxy for feedyard cull prices (Horton et al., 2021). Cattle prices were matched with the existing data set based on placement date and harvest date. Weekly corn prices, obtained from the LMIC, served as a proxy for cost of feed. Corn prices were matched with the existing data based on placement date, assuming that feed was purchased prior to placement. Annualized interest rates were obtained from the Federal Reserve Bank of Kansas City (2024).

Building upon methods from Dennis et al. (2020) and Dennis et al. (2018), estimated net returns (NR) to cattle feeding after an AIP diagnosis were determined by:


$$\begin{array}{lll} NR=\; & TR-FDRC-FC-YC-IC-\; & \left(TxC\cdot Tx\right)-\left(ChC\cdot Tx\right) \end{array}$$ (1)

where *TxC* is the cost of treatment ($23.60/animal), *Tx* is number of treatments, and *ChC* is chute cost ($1.50/animal). Total revenue (*TR*) and feeder cattle cost (*FDRC*) were estimated using pen-level data, defined as:


$$\begin{array}{l} TR=DP\cdot avgcarcasswt \end{array}$$ (2)

$$\begin{array}{l} FDRC=FRP\cdot CPW \end{array}$$ (3)

where *DP* is dressed carcass price ($/kg), *FRP* is feeder cattle price ($/kg), and *CPW* is feeder cattle purchase weight. A dressing percentage of 64% was assumed to calculate average carcass weight (*avgcarcasswt*) from shipping weight. Yardage cost (*YC*) and interest cost (*IC*) were defined as:


$$\begin{array}{l} YC=0.4\cdot DOF \end{array}$$ (4)

$$\begin{array}{l} IC=\left(0.5\cdot \left(YC+FC\right)+FDRC\right)\cdot DOF\cdot \left(\frac{IR}{365}\right) \end{array}$$ (5)

where *DOF* is estimated days on feed and *IR* is annualized interest rate. Because many cattle in our data do not finish with their cohort, average feeding cost is estimated by:


$$\begin{array}{l} FC=FEED\cdot \left(AFC\cdot \left(CSW-CPW\right)\right)\;\;\; \end{array}$$ (6)

where *FEED* is the price of corn ($/kg), *AFC* is average feed conversion, and *CSW* is an adjusted individual finishing weight. *CSW* is estimated by:


$$\begin{array}{l} CSW=TreatmentWeight+\left(ADG_{T}\cdot DaystoHarvest\right)\; \end{array}$$ (7)

where *TreatmentWeight* is the weight recorded at AIP treatment^1^, *ADG*_*T*_ is the estimated average daily gain after AIP treatment, and *DaystoHarvest* is estimated days on feed after AIP treatment. The difference in weight gain between AIP treatments for cattle that received more than one AIP treatment was used to determine an average *ADG*_*T*_ for cattle that received any number of AIP treatments.

Estimated net returns, as described in equations (1-7), offer an approximation of value for an animal with a given treatment outcome. However, expected net returns can provide long-term insights by accounting for the probability of different treatment outcomes. The expected net return is different than the estimated net return, in that the expected net return is determined by computing the sum of the products of estimated net returns for terminal outcomes and probability of occurrence of each outcome from the data distribution.

A simple linear mixed model and Wald Chi-Square test was implemented to test the association of sex, placement weight, and number of AIP treatments with whether an animal finishes with its cohort. A p-value ≤ 0.05 indicated that sex, placement weight, and number of AIP treatments were statistically associated with whether an animal finishes with its cohort. While not central to the findings of this study, this analysis validates the relevance of sex, placement weight, and number of treatments in the examination of net returns for feedyard cattle identified with AIP.

## RESULTS

**Figure 3.**
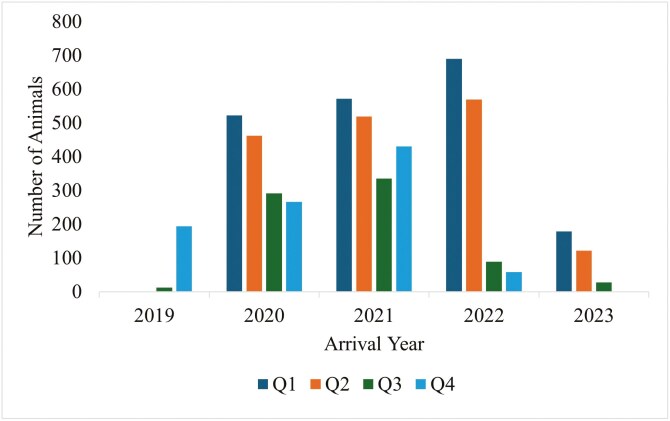
Number of Central U.S. high plains feedyard cattle placed each quarter from 2019 through 2023.

Table 1 provides mean descriptive statistics generated for performance variables used in net return estimation for cohorts of feedyard cattle that had at least one animal identified for treatment with AIP. These data consisted of beef heifers (*n* = 3,483) and steers (*n* = 1,496) from 9 Central U.S. high plains feedyards. The average capacity of the contributing feedyards ranged from 15,000 to 35,000. Animals included in this analysis were placed on feed from 2019 through 2023 (Figure 3). On average, cattle spent 132 DOF, gaining 1.56 kg/day at 6.14 kg feed/kg gain. The average pen mortality was 4.1% and 3.3% for heifers and steers, respectively.

**Table 1.** Mean descriptive statistics of performance variables for cohorts from which Central U.S. high plains feedyard cattle with acute interstitial pneumonia (AIP) were identified, overall and stratified by sex

Variable	All	Heifers	Steers
Estimated Days on Feed	132	129	139
Placement Weight, kg	336	330	351
Harvest Weight, kg	581	562	629
Morbidity, %	24.97	25.59	23.39
Mortality, %	3.89	4.10	3.33
Average Daily Gain, kg	1.56	1.51	1.70
Average Feed Conversion, kg feed/kg gain	5.35	5.42	5.16
Number of animals identified with AIP	5,339	3,483	1,496

**Figure 4.**
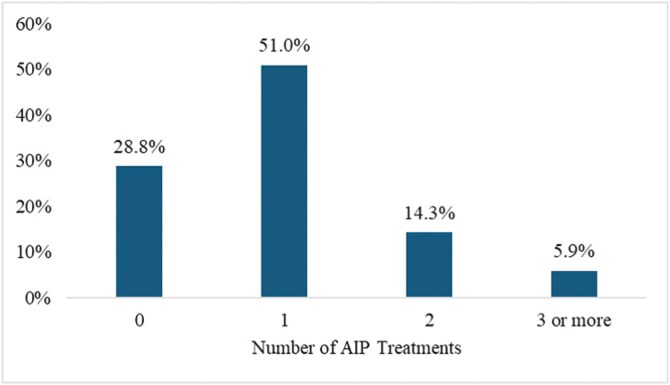
Percentage of Central U.S. high plains feedyard cattle identified with acute interstitial pneumonia (AIP) receiving zero, one, two, and three or more AIP treatments.

Of the cattle identified with AIP (*n* = 5,339), approximately 50% died in the feedyard, and 29% of cattle with a designated AIP event were found dead in the pen and never received treatment for AIP (Figure 3). Of the cattle that were found dead in the pen, approximately 35% were previously treated for BRD. Approximately 50% of the cattle identified with AIP were treated once, 14% were treated twice, and 6% were treated three or more times (Figure 4).

Most cattle identified as morbid with AIP received the first treatment within 50 d of expected harvest regardless of placement weight. Similarly, cattle that were found dead in the pen and designated as an AIP case were, on average, identified around 25 d prior to expected harvest. Nearly 25% of cattle identified with AIP were previously treated for BRD. Of those cattle previously treated for BRD and later identified with AIP, 21% finished with their cohort, 56% died, and 23% were culled.

Costs associated with cattle feeding varied modestly by sex (Table 2). Feeder cattle cost was higher for steers compared to heifers, likely due to heavier weight at purchase. On average, steers had greater DOF, and therefore had higher yardage and interest costs. However, feed costs were lower for steers than heifers, likely due to better performance (higher ADG, lower AFC). The dressed carcass price received was $4.65/kg for heifers and $4.56/kg for steers. Carcass information was not available for the cattle in this study; therefore, yield grade and quality grade premiums and discounts were not accounted for in net return estimation. Due to higher costs of feeding and lower dressed prices, it was anticipated that average net return could be considerably lower for steers placed during the time period evaluated in this study (2019 to 2023).

**Table 2.** Mean descriptive statistics of economic variables used in net return estimation for Central U.S. high plains feedyard cattle identified with acute interstitial pneumonia (AIP), stratified by sex

Variable	All	Heifers	Steers
Feeder Cattle Cost, $/animal	1,048.13	1,001.59	1,167.68
Feed Cost, $/animal	274.32	273.10	277.47
Yardage Cost, $/animal	52.82	51.67	55.75
Interest Cost, $/animal	24.87	23.16	29.28
Dressed Carcass Price, $/kg	4.63	4.65	4.56
Number of animals identified with AIP	5,339	3,483	1,496

**Figure 5.**
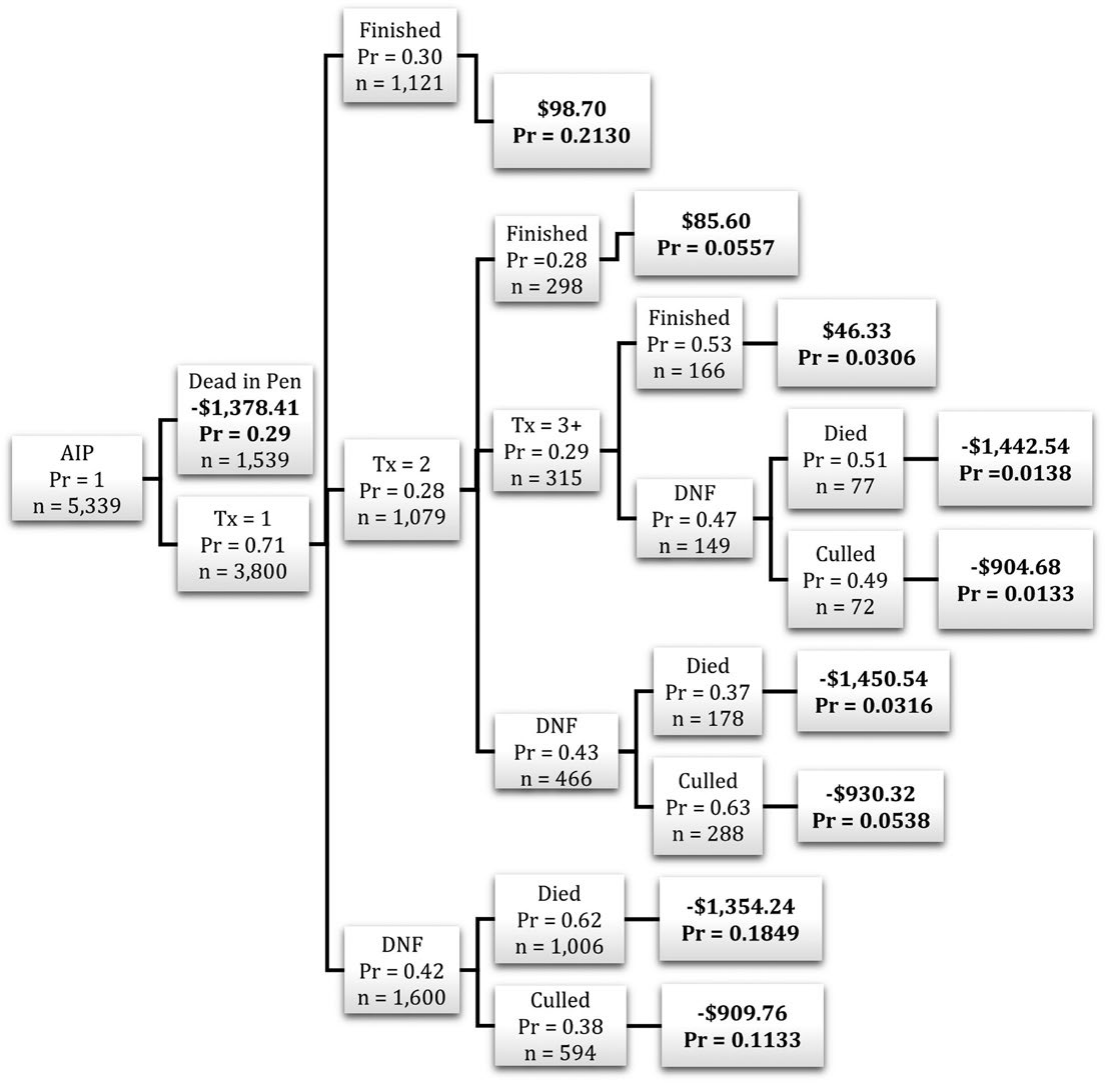
Decision Tree Framework describing probability of outcome occurrence and estimated net return for Central U.S. high plains feedyard cattle identified with acute interstitial pneumonia (AIP). Dead in Pen = never treated for AIP; Tx = treatment; Finished = harvested with cohort; DNF = Did Not Finish; Died = died prior to harvest; Culled = culled prior to expected harvest; Pr = within data probability of each outcome.

Estimated net returns for various outcomes of cattle identified with AIP during the feeding phase are presented in Figure 5. On average, estimated net returns were positive for cattle that finished with their cohort even after multiple treatments, although estimated net returns were lower for cattle that received a greater number of AIP treatments. Estimated net returns were $98.70/animal for cattle that finished with their cohort after one treatment, $85.60/animal for those that finished after two treatments, and $46.33/animal for those that finished after three or more treatments.

Of the cattle identified with AIP, 29% were found dead in the pen (identified postmortem) and never received treatment. No cattle were identified with AIP and culled without receiving at least one AIP treatment. Of the cattle treated once for AIP, approximately 30% recovered and finished with their cohort without further treatment for AIP (Figure 5). Of those treated once, approximately 28% relapsed and were treated at least one more time, while 42% did not remain in the feedyard. Of the 42% that did not finish after the first AIP treatment, 62% died and 38% were culled. The probability of not finishing was greater for cattle receiving a second AIP treatment, and even greater for cattle receiving a third AIP treatment.

**Figure 6.**
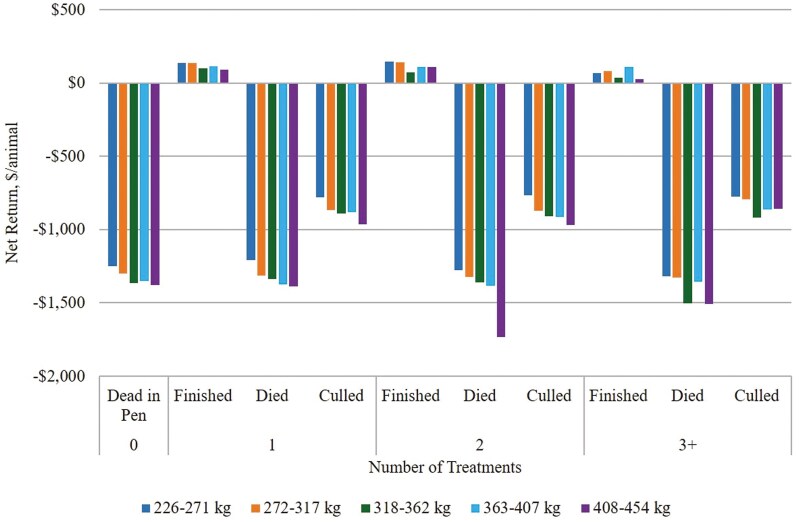
Estimated net returns for heifers (n = 3,843) from Central U.S. high plains feedyards identified with acute interstitial pneumonia (AIP). Finished = harvested with cohort; Died = died prior to harvest; Culled = culled prior to expected harvest.

**Figure 7.**
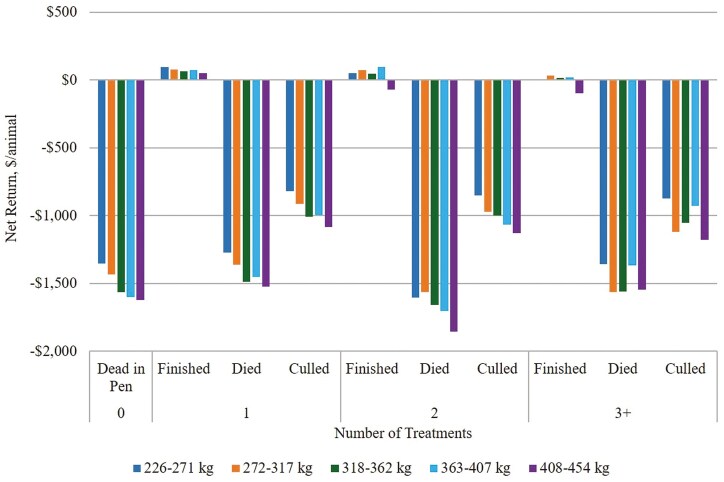
Estimated net returns for steers (n = 1,496) from Central U.S. high plains feedyards identified with acute interstitial pneumonia (AIP). Finished = harvested with cohort; Died = died prior to harvest; Culled = culled prior to expected harvest.

Estimated returns for cattle identified with AIP vary by sex, placement weight, and number of AIP treatments (Figure 6; Figure 7). On average, heifers that finish after AIP treatment were estimated to bring positive returns across all placement weights. Heifers placed between 226 to 271 kg that finished after two AIP treatments were estimated to bring the highest net return of $147.17/animal, on average (Figure 6, Table A.1.). Heifers that finished after three or more AIP treatments were generally estimated to bring higher returns if placed at heavier weights. There was little variation in estimated net returns from culling heifers after one or two AIP treatments; however, estimated net returns from culling heifers were slightly higher following three or more AIP treatments. Estimated economic losses were greatest for heifers placed between 408 to 454 kg that died after two AIP treatments. Steers that finished after two treatments and were placed between 363 to 407 kg were estimated to bring the highest return at $96.60/animal (Figure 7, Table A. 2.). Steers placed between 408 to 454 kg and treated more than once for AIP were not estimated to bring positive returns, even if they recovered and finished with their cohort. Similar to heifers, there was little variation in estimated net returns from culling steers after one or two AIP treatments. Estimated economic losses were greatest for steers that die after two AIP treatments.

Probabilities of treatment outcomes from the data distribution were used to calculate expected returns for cattle identified with AIP at each decision node. The expected net return to feeding healthy cattle was $193.67/animal, and the expected net return for cattle identified with AIP was -$871.19/animal. Expected returns were greater, on average, for cattle receiving more AIP treatments. The expected net return was -$639.71/animal for cattle treated once for AIP, -$612.41/animal for those treated twice for AIP, and -$529.57/animal for those treated three or more times for AIP.

## DISCUSSION

These data represented beef heifers (*n* = 3,483) and steers (*n* = 1,496), which is consistent with previous studies finding that AIP tends to occur more often in heifers (Ayroud et al., 2000; Loneragan et al., 2001a; Valles et al., 2016). The majority of cattle treated for AIP received the first AIP treatment within 50 d of expected harvest, which is consistent with previous studies of AIP in cattle feedyards (Loneragan & Gould, 2000; Loneragan et al., 2001a, 2001b; Bortoluzzi et al., 2023). This analysis offers insights for returns from cattle identified with AIP on the basis of sex, placement weight, and number of AIP treatments. Estimated net returns were lower for steers treated for AIP compared to heifers, regardless of placement weight and number of treatments. This is likely attributed to higher investment and lower dressed carcass prices for steers during this time period. As expected, estimated net returns were generally lower for cattle receiving more AIP treatments and cattle placed in the feedyard at heavier weights. Estimated net returns remained positive for cattle that were successfully treated for AIP and finished with their cohort, with the exception of steers placed between 408 to 454 kg that were treated more than once for AIP. On average, estimated returns were $98.70/animal for cattle that finished with their cohort after one AIP treatment, $85.60/animal for those that finished after two AIP treatments, and $46.33/animal for those that finished after three or more AIP treatments. Although net returns were estimated to remain positive for cattle that finish with their cohort after multiple AIP treatments, the probability of each outcome should be considered when making the decision to cull or treat cattle that have been identified with AIP. The expected net return for cattle identified with AIP (-$853.83/animal) was considerably lower than the expected net return from healthy feedyard cattle ($193.67/animal).

Cattle culled after an AIP diagnosis were not estimated to bring positive net return, even if culled after the first treatment. Negative returns for culls are likely due to increased feeding costs for animals culled late in the feeding period. Decisions to cull or keep cattle in the feedyard after treatment are largely based on cull prices. If expected return from treating the animal is greater than estimated return from culling, the animal should remain in the feedyard. In this analysis, expected returns are greater for cattle treated for AIP compared to cattle that are culled before or after AIP treatment. For example, expected net returns for cattle treated once for AIP were -$639.71/animal, while estimated net returns from culling after one AIP treatment were -$909.76/animal. Similarly, expected net returns were -$612.41/animal from cattle treated twice for AIP and -$529.57/animal for those treated three or more times, while estimated net returns from culling were -$930.32/animal and -$904.68, respectively. Although largely negative, expected net returns from keeping and treating cattle identified with AIP were still greater than estimated net return from culling at each decision point, implying that cattle identified with AIP should not be culled extemporaneously. However, returns from culls are greater than returns from animals that died in the feedyard, suggesting that culling would be preferable in some cases. The decision to cull versus treat an animal should incorporate the probability of treatment success for that particular animal. While not included in this analysis, additional factors such as individual and cohort risk level, previous illness, and treatment timing should be considered in culling decisions.

Although data were based on field observations from U.S. Central high plains feedyards, we have demonstrated that economic returns from cattle identified with AIP vary across sex, placement weight, and treatment outcome. However, this analysis is not without limitations. Chute-side diagnosis of AIP is not perfect, and consideration of other health indicators and risk factors not evaluated in this study may contribute to the decision to cull cattle identified with AIP. Metaphylaxis treatment and incoming health status were not included in the data; therefore, we are unable to evaluate differences in returns from high-risk and low-risk cattle treated for AIP. A more detailed look at the effects of treatment timing on treatment outcomes may be beneficial for producers deciding whether to treat or cull cattle identified with AIP. The majority of feedyard cattle identified with AIP are first treated for AIP within 50 d of expected harvest, and only 30% of cattle in this data, excluding those found dead in the pen, recovered and finished after the first AIP treatment. A greater understanding of factors associated with the probability of first AIP treatment success is imperative for producers making decisions regarding management of feedyard cattle identified with AIP. Association between expected AIP treatment outcomes and prior disease management is another area for future analysis. In our data, approximately 25% of cattle identified with AIP were previously treated for BRD, of which 56% died and 23% were culled. Cohort and individual demographics such as BRD morbidity may provide more insight into the risk associated with prior respiratory diagnoses and AIP treatment outcomes. Further analysis with predictive modeling of economic and treatment outcomes are necessary for determining best management of AIP cases identified in cattle feedyards.

## Supplementary Material

txaf091_suppl_Supplementary_Materials_1

## Footnotes

1 The last recorded treatment weight is used to estimate CSW. Cull weight and death weight are estimated in a similar manner, using last recorded treatment weight and estimated days to cull or death.
