# Supplementary material for: Economic outcomes associated with acute interstitial pneumonia in Central U.S. High Plains feedyards
Source: Transl Anim Sci. 2025 Jul 11;9:txaf091. doi: 10.1093/tas/txaf091 (PMC12342467; doi:10.1093/tas/txaf091)
Supplement: txaf091_suppl_Supplementary_Materials_1 [file txaf091_suppl_supplementary_materials_1.docx]

| **Table A.1.** Average Net Returns for Heifers Treated for AIP | | | | | | |
| --- | --- | --- | --- | --- | --- | --- |
| Treatment | Outcome | Placement Weight (kg) | | | | |
|  |  | 226-271 kg | 272-317 kg | 318-362 kg | 363-407 kg | 408-454 kg |
| 0 | Dead in Pen | -$1,248.05 | -$1,301.72 | -$1,363.75 | -$1,348.76 | -$1,379.52 |
| 1 | Finished | $136.71 | $136.50 | $102.59 | $114.28 | $90.35 |
|  | Died | -$1,206.58 | -$1,313.13 | -$1,337.16 | -$1,371.84 | -$1,387.80 |
|  | Railed | -$777.87 | -$864.76 | -$889.96 | -$880.51 | -$961.45 |
| 2 | Finished | $147.17 | $144.06 | $73.77 | $111.13 | $110.65 |
|  | Died | -$1,276.77 | -$1,324.83 | -$1,360.03 | -$1,381.44 | -$1,732.90 |
|  | Railed | -$764.73 | -$869.56 | -$906.60 | -$910.77 | -$967.85 |
| 3+ | Finished | $66.83 | $81.73 | $34.30 | $108.34 | $25.82 |
|  | Died | -$1,316.24 | -$1,325.70 | -$1,502.42 | -$1,352.68 | -$1,506.11 |
|  | Railed | -$775.33 | -$794.59 | -$915.99 | -$861.01 | -$856.21 |

| **Table A.2.** Average Net Returns for Steers Treated for AIP | | | | | | |
| --- | --- | --- | --- | --- | --- | --- |
| Treatment | Outcome | Placement Weight (kg) | | | | |
|  |  | 226-271 kg | 272-317 kg | 318-362 kg | 363-407 kg | 408-454 kg |
| 0 | Dead in Pen | -$1,353.22 | -$1,432.39 | -$1,562.07 | -$1,599.75 | -$1,622.97 |
| 1 | Finished | $94.88 | $77.87 | $64.42 | $72.33 | $49.24 |
|  | Died | -$1,272.39 | -$1,363.13 | -$1,488.15 | -$1,449.44 | -$1,522.83 |
|  | Railed | -$818.43 | -$914.80 | -$1,005.89 | -$993.25 | -$1,085.87 |
| 2 | Finished | $50.19 | $74.43 | $44.67 | $96.60 | -$70.77 |
|  | Died | -$1,603.50 | -$1,563.51 | -$1,659.21 | -$1,704.46 | -$1,856.85 |
|  | Railed | -$849.36 | -$971.17 | -$999.31 | -$1,065.50 | -$1,126.53 |
| 3+ | Finished |  | $34.61 | $14.83 | $21.24 | -$97.73 |
|  | Died | -$1,355.88 | -$1,563.64 | -$1,560.28 | -$1,367.16 | -$1,547.10 |
|  | Railed | -$874.97 | -$1,118.84 | -$1,051.18 | -$925.85 | -$1,176.06 |
